# Efficacy of endoscopic dorsal medial branch radiofrequency ablation combined with manual therapy for chronic lumbar facet joint pain

**DOI:** 10.3389/fresc.2026.1806911

**Published:** 2026-07-07

**Authors:** Yangming Hu, Yang Liu, Dian Zhong, Shan Wu, Yang Wang

**Affiliations:** Department of Spine Surgery, The Second Affiliated Hospital of Chongqing Medical University, Chongqing, China

**Keywords:** combination therapy, lumbar facet joint pain, manual therapy, pain management, radiofrequency ablation

## Abstract

**Purpose:**

This study aimed to evaluate the clinical efficacy of combining radiofrequency ablation (RFA) with manual therapy (MT) compared to either modality alone in patients with chronic lumbar facet joint pain.

**Method:**

A retrospective observational study was conducted on 234 patients with chronic lumbar facet joint pain. Patients were divided into three groups: MT alone (*n* = 83), RFA alone (*n* = 77), and combined RFA + MT (*n* = 74). Pain intensity was assessed using the Visual Analog Scale (VAS), functional disability was measured with the Roland-Morris Questionnaire (RMQ) and Oswestry Disability Index (ODI), and health-related quality of life was evaluated using the Short-Form 36 (SF-36) questionnaire. Assessments were performed at baseline and at 1, 3, and 6 months post-treatment.

**Result:**

The three groups were well-matched at baseline. At all follow-up intervals, the RFA + MT group demonstrated significantly greater improvements compared to both monotherapy groups (*p* < 0.001). The RFA + MT group achieved the lowest (best) scores in VAS (3.2 ± 0.6 at 6 months), RMQ (8.2 ± 1.5), and ODI (19.0 ± 2.8) at the 6-month follow-up. Furthermore, the RFA + MT group showed significantly superior outcomes in all eight domains and the total score of the SF-36 survey at 6 months, indicating a comprehensive enhancement in quality of life. Minor transient adverse events occurred in 3 patients (4.1%) in the RFA + MT group, resolving within one month, with no serious complications reported in any group.

**Conclusion:**

The combination of radiofrequency ablation and manual therapy was associated with significantly greater improvements compared to either treatment alone in reducing pain, improving functional capacity, and enhancing the quality of life in patients with chronic lumbar facet joint pain over a 6-month period. This combined approach represents a promising synergistic strategy for managing this condition.

## 1 Introduction

Lumbar facet joint pain is a common disease, with approximately 80% of people experiencing low back pain during their lifetime (1, 2). This condition more commonly affects women than men. Although persistent back pain can arise from various causes including intervertebral discs, ligaments, facet joints, and nerve dysfunction, facet joint dysfunction is recognized as a critical factor in the development of chronic low back pain. The prevalence of lumbar facet joint pain varies significantly, ranging from 15% to 45% (3, 4). Characterized by spinal and paravertebral pain, lumbar facet joint pain typically presents without neurological symptoms. Acute lumbar facet joint pain, defined as lasting less than 1 month, is considered a self-limiting condition that generally requires no treatment. Recurrent pain persisting beyond 3 months after onset is classified as chronic lumbar facet joint pain. While acute episodes of low back pain usually do not significantly impair work or daily activities, persistent back pain—particularly from lumbar facet joints—can lead to severe mobility restrictions, increased disability rates, and higher healthcare costs (5). Therefore, the primary treatment goals for lumbar facet joint pain focus on alleviating pain and enhancing quality of life (6).

Radiofrequency ablation (RFA) is a minimally invasive interventional technique that selectively disrupts nociceptive signaling by applying thermal energy to the medial branch nerves innervating lumbar facet joints (7). As a well-established modality for chronic facet-mediated pain, RFA provides sustained analgesia in 60%–80% of appropriately selected patients for 6–12 months, though diminished efficacy over time remains a limitation (8). Conversely, manual therapy, encompassing spinal mobilization and soft tissue techniques, aims to restore joint mobility, reduce muscle hypertonicity, and modulate pain through biomechanical and neurophysiological mechanisms. Evidence supports its short-term benefits in improving functional outcomes for spinal disorders, particularly when combined with exercise protocols (9–11).

While both modalities demonstrate standalone utility, their synergistic potential remains underexplored. Mechanistically, RFA-induced pain reduction may enhance patient tolerance for manual therapy, whereas targeted joint mobilization could address residual biomechanical dysfunction perpetuating facet joint overload. Despite theoretical complementarity, few rigorous studies have evaluated combined RFA and manual therapy for facet joint disorders. Existing literature predominantly examines monotherapies or multimodal approaches pairing RFA with pharmacotherapy (12–16). This paucity of evidence highlights a critical knowledge gap regarding optimized integration of structural intervention and neuromodulation.

The present study therefore investigates the clinical efficacy of combined RFA and manual therapy vs. either modality alone, hypothesizing that this dual approach will yield superior pain relief, functional recovery, and sustained benefits in patients with confirmed lumbar facet joint syndrome.

## 2 Method

This was a retrospective observational study and was conducted between January 2024 and December 2024 at the Department of Spine Surgery of The Second Affiliated Hospital of Chongqing Medical University. In this study, 234 patients with lumbar facet joint pain were reviewed. Patient grouping was determined by treatment received as documented in the electronic medical record system: patients who received manual therapy alone were assigned to the MT group, those who received radiofrequency ablation alone were assigned to the RFA group, and those who received both manual therapy and radiofrequency ablation were assigned to the RFA + MT group. Patient data were extracted from electronic medical records of patients who had already completed treatment and follow-up by the time of study initiation. The study was conducted in accordance with the principles of the Declaration of Helsinki and the guidelines of The Second Affiliated Hospital of Chongqing Medical University. Informed consent for the use of de-identified clinical data for research purposes was obtained retrospectively from all individual participants included in the study during follow-up visits or via telephone contact. Data collection was completed in June 2025, which served as the cutoff date for the retrospective analysis.

Inclusion criteria were: age ≥ 18 and ≤60 years low back pain persisting for ≥3 months in duration; understood the treatment plan and able to provide written informed consent; no neurological symptoms; focal tenderness over one or more facet joints; persistent low back pain, hip pain without radicular syndrome, imaging features of facet joint degeneration at the corresponding spinal levels on MR or CT.

Exclusion criteria were: age < 18 or >60 years; focal neurological signs or symptoms; lumbar radicular pain; local or systemic active infection; coagulopathy or other bleeding disorders; surgical segments or adjacent segments with >II°spondylolisthesis, lumbar spondylolisthesis; Cobb angle > 10°; BMI > 34 kg/m²; allergy to local anesthetics; severe clinical depression, anxiety, or any psychotic mental health conditions; potential pregnancy or other contraindications for fluoroscopy use.

**Figure 1:**
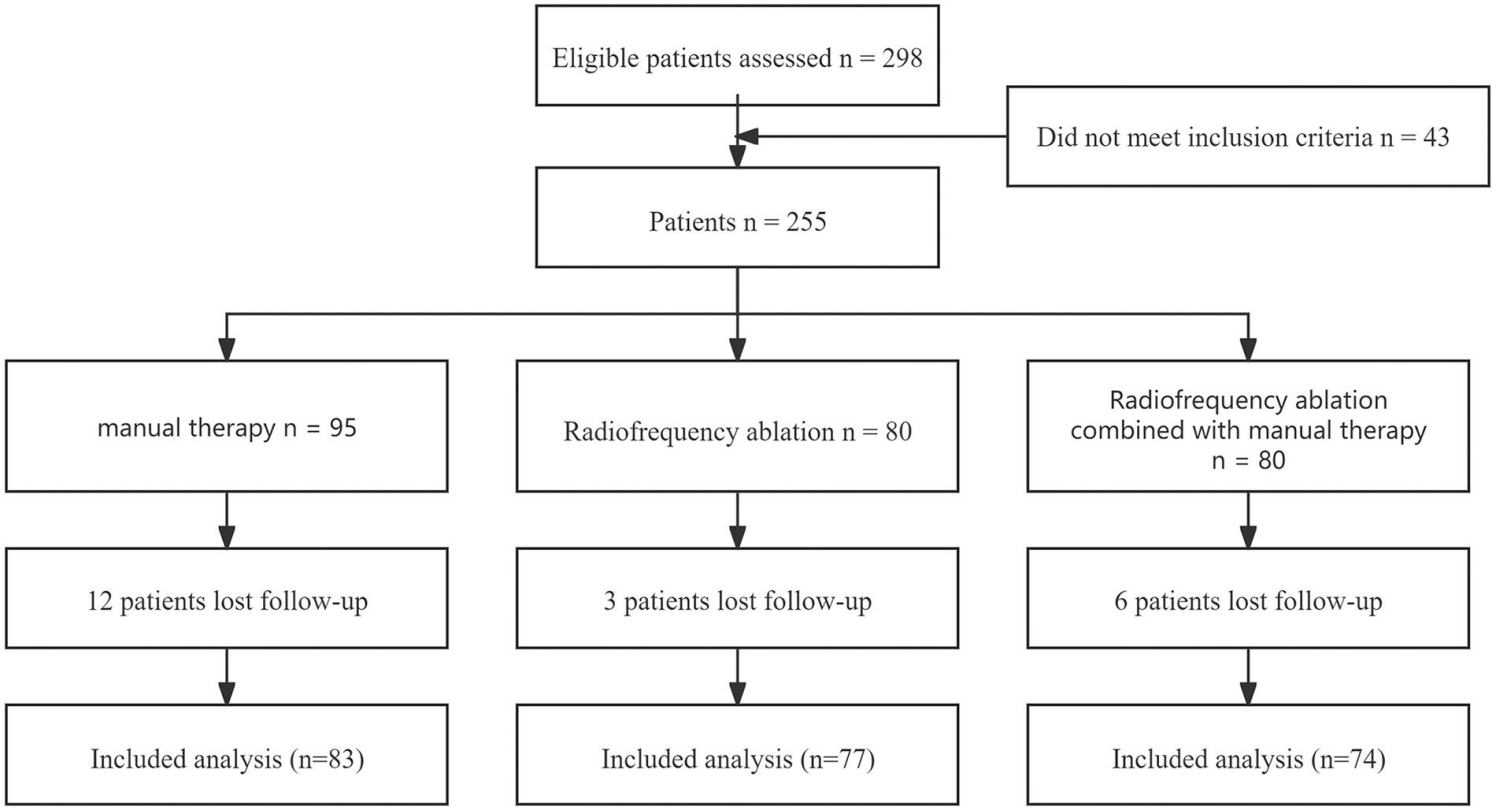
Schematic presentation of participant flow at the 6-month follow-up.

64 patients were excluded from the study because of pain symptoms lasting <3 months (*n* = 23), age < 18 or >60 years (*n* = 16), BMI > 34 (*n* = 4) and lost follow-up (*n* = 21). A total of 234 patients were included in the study (Figure 1).

### 3.1 Measurements

All the selected patients were divided into the MT group, the RFA group and RFA + MT group. Pain assessment and quality of life were measured using a visual analog scale (VAS) and Short-Form36 (SF-36) questionnaire, respectively. Pain was assessed using the VAS, which is a reliable measure of subjective phenomena of various qualities of pain. The SF-36 questionnaire contains eight items including physical functioning (PF), role limitation physical (RP), bodily pain (BP), general health (GH), vitality (VT), Social Functioning (SF), role of emotion (RE), and mental health (MH). SF-36 has previously been validated for patients with chronic non-malignant pain. The Roland-Morris questionnaire (RMQ) and the Oswestry disability index questionnaire (ODI) were used to assess lumbar function. Patients were evaluated using the SF-36 questionnaire, VAS, RMQ and ODI, and administered either as clinician-administered questionnaire or as a mailed questionnaire, before therapy (baseline), and at 1, 3 and 6 months after therapy.

### 3.2 Technique

**Figure 2:**
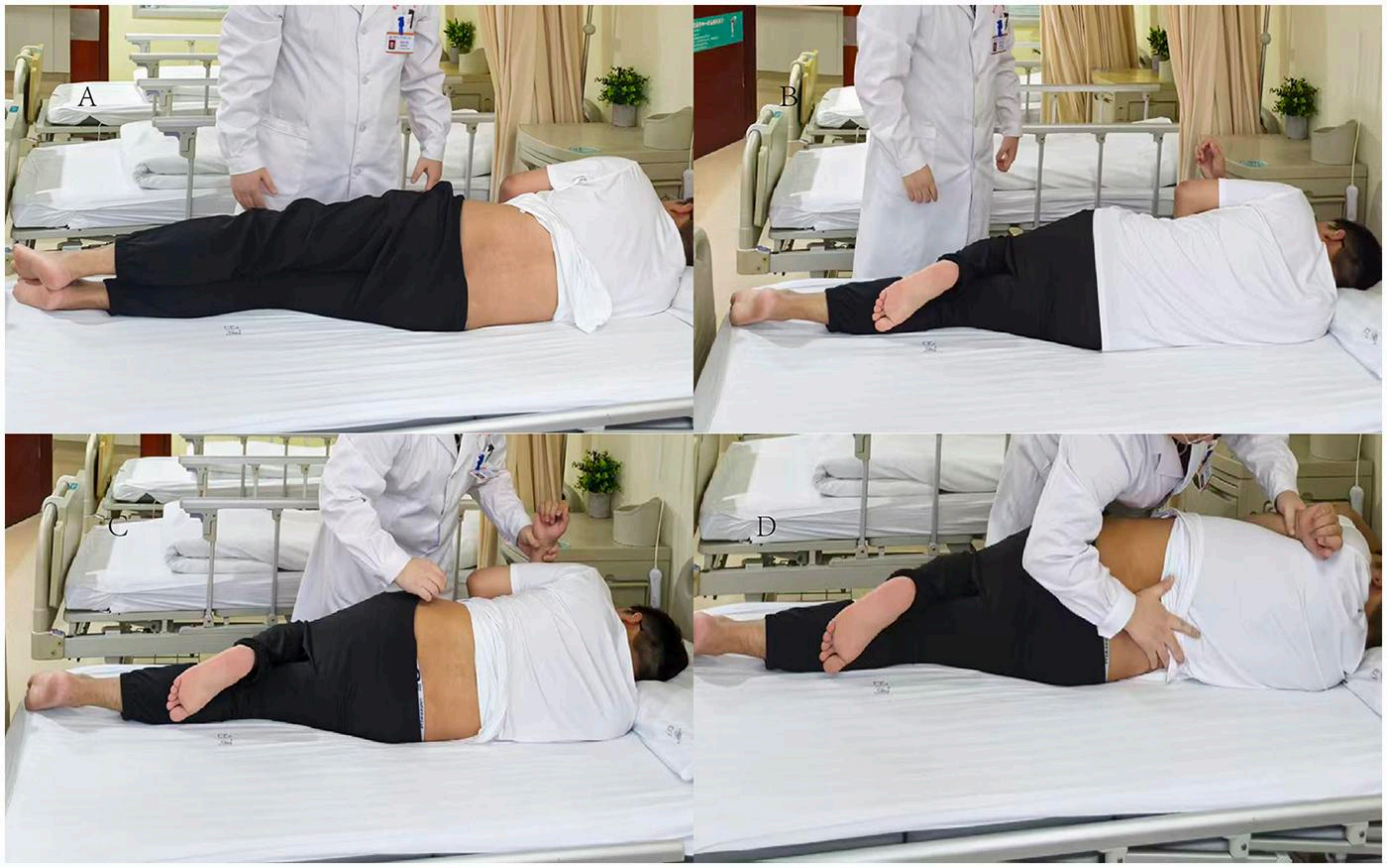
Procedure of MT for a lumbar facet joint pain’ patient. **(A)** preoperative the patient is positioned in left lateral recumbency with the lumbar spine in neutral alignment. **(B)** preoperative the upper hip and knee are flexed to 90° and supported on the table to stabilize the pelvis and create segmental isolation. **(C)** preoperative the therapist's cranial forearm is placed anterior to the shoulder girdle to provide caudal counter-pressure. **(D)** preoperative the caudal forearm contacts the ipsilateral articular process of the target segment. After pre-tensioning to the elastic barrier, a high-velocity, low-amplitude thrust of approximately 5° of rotation is delivered in a cephalad–ventral direction, frequently accompanied by an audible cavitation.

MT: The patient lies in a side-lying position with the upper leg's hip and knee flexed at 90°. The therapist presses one forearm on the patient's shoulder and places the other forearm against the articular process. They apply force in opposite directions until a barrier is felt, then deliver a quick, small thrust (about 5° of rotation). A click may be heard, marking the end of the treatment (Figure 2). Manual therapy was performed by a single spine surgeon (YW) with more than 10 years of experience in spinal surgery and manual therapy. Each patient received 2 sessions per week for 4 weeks (total 8 sessions), with each session lasting approximately 20–30 min. All manual therapy sessions were conducted by the same surgeon to ensure maximal consistency in technique delivery. The frequency, duration, and number of sessions were uniform across all patients in the MT and RFA + MT groups.

**Figure 3:**
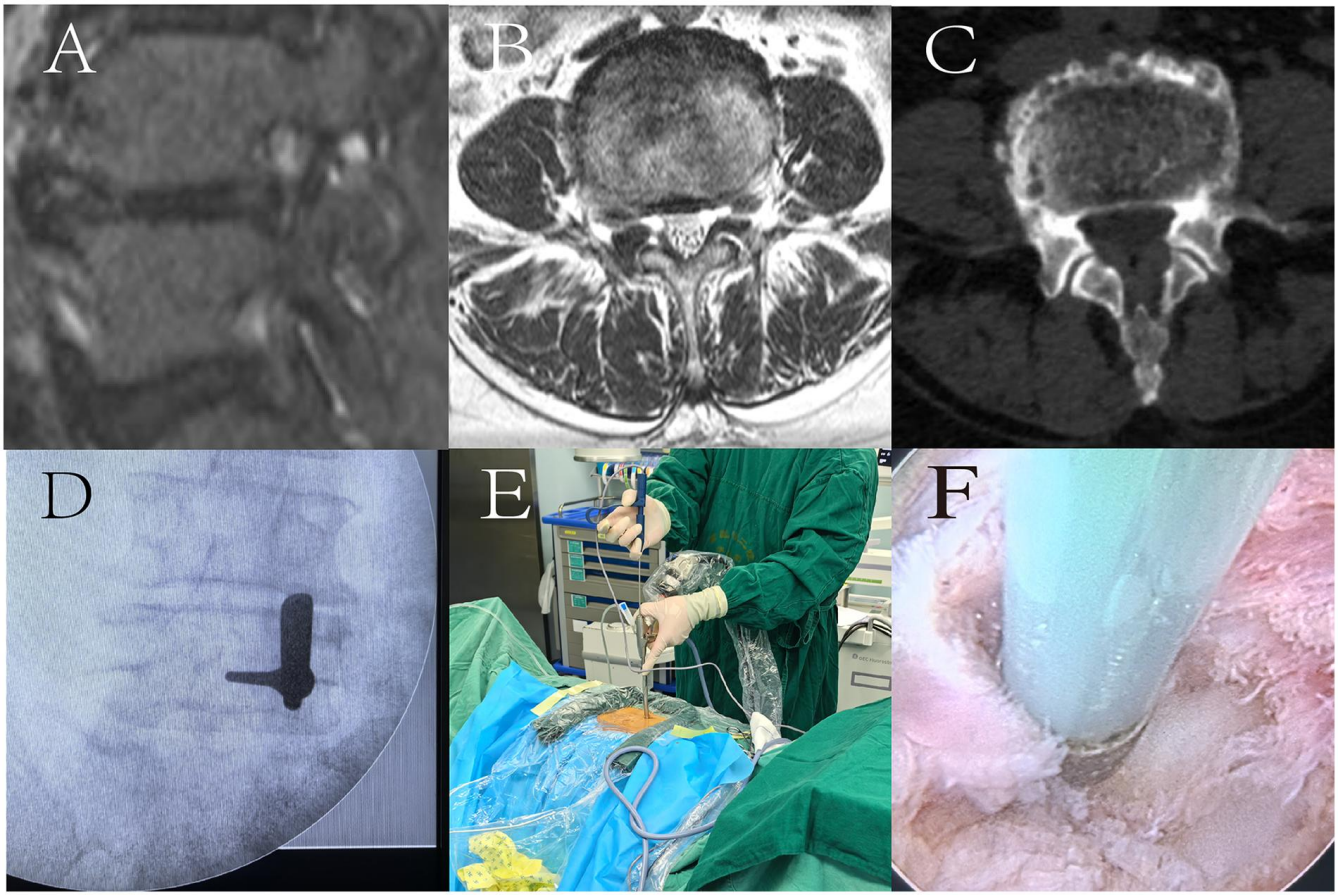
Surgical procedure of RFA and preoperative imaging of patients. **(A–C)** preoperative MRI and CT showed L4/5 facet joint degeneration. **(D–F)** D image showed the anteroposterior fluoroscopy utilized during the RFA procedure. Image E showed the RFA working channel. Image F demonstrated the nerve following RFA.

RFA: The patient was placed in a prone position. 0.5 mL of 2% lidocaine was injected to the dorsal medial branch at the level of the facet joint. Patients with at least a 50% reduction in VAS score measured 30 min after injection were eligible for the next step. Under the guidance of endoscopic, a puncture needle was advanced into the origin of the transverse and the superior articular process. On the x-ray orthopantomography, the needle tip is situated at the junction of the outer edge of the superior articular eminence and the superior border of the transverse eminence (Figure 3). After confirmation of proper location, radiofrequency ablation is performed. Radiofrequency ablation was performed using a standard radiofrequency generator with a 22-gauge, 100 mm radiofrequency cannula with a 5 mm active tip. Continuous radiofrequency was applied at 80 °C for 90 s per level after sensory (50 Hz) and motor (2 Hz) stimulation confirmed proper needle placement. A single treatment session was performed per spinal level. All RFA procedures were conducted by one attending spine surgeons (YW) with more than 10 years of experience in interventional pain procedures to ensure technical consistency.

### 3.3 Statistics

Comparisons were performed using chi-square tests or Fisher's exact tests, for categorical variables and analysis of variance (ANOVA). for continuous variables. All analyses were performed using SPSS Statistics 27.0 (IBM, Armonk, NY). For all analyses, *p*-value < 0.05 was considered statistically significant.

## 4 Result

A total of 234 patients were included in this study, distributed across three treatment groups: MT (*n* = 83), RFA (*n* = 77), and RFA + MT (*n* = 74). The demographic and clinical characteristics of the patients are summarized in Table 1. There were no statistically significant differences among the three groups in terms of age, sex, duration of pain, or treated spinal level. The mean age was 52.2 ± 9.2 years in the MT group, 50.2 ± 10.9 years in the RFA group, and 51.3 ± 10.6 years in the RFA + MT group (*p* = 0.592). Sex distribution was also comparable across groups (*p* = 0.669), with male patients comprising 36.5%, 45.5%, and 47.0% of the MT, RFA, and RFA + MT groups, respectively. The mean duration of pain was similar among groups, with values of 4.3 ± 1.5 months, 4.3 ± 1.3 months, and 4.4 ± 1.4 months for the MT, RFA, and RFA + MT groups, respectively (*p* = 0.784). The distribution of treated spinal levels (L4/5 and L5/S1) did not differ significantly among groups (*p* = 0.853). These results indicate that the three treatment groups were well-matched at baseline, with no significant differences in demographic or clinical characteristics.

**Table 1:** Patient demographics.

Variable	MT	RFA	RFA + MT	*p*
Total(*n*)	83	77	74	
Age(years)	51.3 ± 10.6	50.2 ± 10.9	52.2 ± 9.2	0.592
Sex(*n*)				0.669
Male	39	35	27	
Female	44	42	47	
Duration of pain(months)	4.3 ± 1.5	4.3 ± 1.3	4.4 ± 1.4	0.925
Level(*n*)				0.823
L2/3	0	0	1	
L3/4	3	2	3	
L4/5	51	47	49	
L5/S1	29	28	21	

### 4.1 VAS

Preoperative VAS scores were comparable across all three groups (MT: 7.2 ± 1.3; RFA: 7.2 ± 1.1; RFA + MT: 7.3 ± 1.2; *p* = 0.831), indicating no significant baseline differences in pain levels. Postoperatively, significant differences emerged among the groups at all follow-up time points (*p* < 0.001). At 1 month, the RFA + MT group reported the lowest pain scores (3.8 ± 0.7), followed by the RFA group (4.1 ± 0.7), with the MT group reporting the highest scores (5.1 ± 0.7). This trend continued at the 3- and 6-month assessments, with the RFA + MT group maintaining the most substantial pain reduction (3.3 ± 0.6 and 3.2 ± 0.6, respectively). The mean change in VAS score from preoperative to 6 months (*Δ*VAS) also differed significantly between groups (*p* < 0.001), with the greatest improvement observed in the RFA + MT group (*Δ* = 4.1 ± 0.7), exceeding the established minimal clinically important difference (MCID) of 1.5–2.0 points for VAS in chronic low back pain (Table 2).

**Table 2:** Comparison of PROMs.

PROMs	MT	RFA	RFA + MT	*P* value
VAS				
Preoperative (Mean ± SD)	7.2 ± 1.3	7.2 ± 1.1	7.3 ± 1.2	0.831
Postoperative (1M)	5.1 ± 0.7	4.1 ± 0.7	3.8 ± 0.7	<0.001
Postoperative (3M)	5.7 ± 0.8	3.7 ± 0.8	3.3 ± 0.6	<0.001
Postoperative (6M)	5.6 ± 0.8	3.6 ± 0.8	3.2 ± 0.6	<0.001
*Δ* (Preoperative to 6M)	1.6 ± 1.0	3.6 ± 1.0	4.1 ± 0.7	<0.001
RMQ				
Preoperative (Mean ± SD)	14.8 ± 2.0	14.8 ± 2.0	15.1 ± 2.0	0.549
Postoperative (1M)	11.4 ± 0.8	9.4 ± 1.1	9.0 ± 1.8	<0.001
Postoperative (3M)	10.9 ± 1.0	8.9 ± 1.1	8.3 ± 1.4	<0.001
Postoperative (6M)	10.7 ± 1.1	8.7 ± 1.1	8.2 ± 1.5	<0.001
ODI				
Preoperative (Mean ± SD)	31.2 ± 2.7	31.2 ± 2.7	31.4 ± 2.9	0.870
Postoperative (1M)	26.8 ± 2.0	23.8 ± 3.0	23.0 ± 2.8	<0.001
Postoperative (3M)	26.9 ± 2.6	20.9 ± 2.1	19.9 ± 2.4	<0.001
Postoperative (6M)	26.1 ± 2.5	20.1 ± 2.2	19.0 ± 2.8	<0.001

VAS, visual analog scale; RMQ, roland-morris questionnaire; ODI, oswestry disability index.

### 4.2 RMQ

Preoperative RMQ scores showed no significant intergroup differences (MT: 14.8 ± 2.0; RFA: 14.8 ± 2.0; RFA + MT: 15.1 ± 2.0; *p* = 0.549). Postoperative RMQ scores demonstrated significant improvement in all groups, with statistically significant differences among them at 1, 3, and 6 months (*p* < 0.001). The RFA + MT combination group consistently achieved the lowest (best) scores at each postoperative follow-up (9.0 ± 1.8 at 1M, 8.3 ± 1.4 at 3M, and 8.2 ± 1.5 at 6M), indicating superior improvement in back-specific disability compared to the single-modality groups. The mean improvement from baseline (15.1 to 8.2, *Δ* = 6.9 points) exceeds the MCID of 2–3 points reported for RMQ in chronic low back pain populations.

### 4.3 ODI

Similarly, baseline ODI scores were well-matched across the groups (*p* = 0.870). Postoperative assessment revealed a significant overall difference between the treatment groups at all time points (*p* < 0.001). The RFA + MT group exhibited the most pronounced functional improvement, achieving the lowest ODI scores at the 1-month (23.0 ± 2.8), 3-month (19.9 ± 2.4), and 6-month (19.0 ± 2.8) evaluations. The mean improvement from baseline (31.4 to 19.0, *Δ* = 12.4 points) meets the established MCID of 10–12 points for ODI in lumbar spine conditions. The RFA-alone group showed better outcomes than the MT-alone group, which consistently reported the highest ODI scores throughout the follow-up period (e.g., 26.1 ± 2.5 at 6 months).

### 4.4 SF-36

At baseline, there were no statistically significant differences among the three treatment groups (MT, RFA, and RFA + MT) across all eight domains and the total score of the SF-36 health survey (all *p* > 0.05). This confirms that the groups were comparable in terms of health-related quality of life prior to receiving therapy.

At the 6-month follow-up, highly statistically significant differences were observed among the groups in all SF-36 domains and the total score (all *p* < 0.001).Physical Function (PF): The RFA + MT group showed the greatest improvement (60.5 ± 5.0), followed by the RFA group (59.5 ± 6.2), with the MT group demonstrating the least improvement (49.6 ± 8.2). Role Physical (RP): The combination RFA + MT group achieved the highest score (57.9 ± 3.8), indicating significantly better outcomes in physical role limitations compared to both the RFA (51.1 ± 8.9) and MT (43.2 ± 9.2) groups. Bodily Pain (BP): Scores were highest in the RFA + MT group (69.4 ± 6.1), followed by the RFA group (64.6 ± 6.3), with the MT group reporting a score of 56.5 ± 7.3. General Health (GH): The RFA and RFA + MT groups reported similar and superior outcomes (60.0 ± 5.8 and 60.5 ± 7.3, respectively) compared to the MT group (53.0 ± 9.8). Vitality (VT): A marked improvement was seen in the RFA + MT group (66.0 ± 7.8), which was substantially higher than the RFA (58.9 ± 7.1) and MT (51.3 ± 6.1) groups. Social Functioning (SF): The RFA + MT group scored highest (63.0 ± 7.6), outperforming both the RFA (57.8 ± 10.9) and MT (50.7 ± 9.3) groups. Role Emotional (RE): The RFA + MT group again showed the most favorable outcome (63.8 ± 7.3), closely followed by the RFA group (62.8 ± 9.3), and then the MT group (56.7 ± 10.1). Mental Health (MH): All groups improved, but the RFA + MT combination therapy resulted in the most significant enhancement in mental health (83.7 ± 4.7) compared to RFA (75.2 ± 5.8) and MT (73.1 ± 8.6) alone. Total SF-36 Score: The overall quality-of-life score was significantly highest in the RFA + MT group (65.6 ± 1.7), followed by the RFA group (61.3 ± 2.9), with the MT group having the lowest total score (54.3 ± 3.6). The clinical relevance of SF-36 domain improvements can also be assessed through established MCID thresholds. For Physical Function (PF), the RFA + MT group improved by 19.2 points (from 41.3 to 60.5), exceeding the MCID of 5–10 points. For Bodily Pain (BP), the improvement of 33.4 points (from 36.0 to 69.4) surpassed the MCID of 8–10 points (Table 3).

**Table 3:** Health-related quality-of-life outcomes at 6 months after therapy.

SF-36 domains	Baseline			*p* value	Follow-up at 6 months			*p* value
	MT	RFA	RFA + MT		MT	RFA	RFA + MT	
PF	41.4 ± 6.2	39.4 ± 6.5	41.3 ± 6.6	0.096	49.6 ± 8.2	59.5 ± 6.2	60.5 ± 5.0	<0.001
RP	34.3 ± 5.9	33.3 ± 6.7	32.5 ± 5.6	0.181	43.2 ± 9.2	51.1 ± 8.9	57.9 ± 3.8	<0.001
BP	36.8 ± 6.5	36.4 ± 7.5	36.0 ± 6.7	0.769	56.5 ± 7.3	64.6 ± 6.3	69.4 ± 6.1	<0.001
GH	37.6 ± 7.1	36.7 ± 7.5	37.6 ± 7.2	0.673	53.0 ± 9.8	60.0 ± 5.8	60.5 ± 7.3	<0.001
VT	39.2 ± 8.0	39.0 ± 8.2	38.9 ± 7.2	0.971	51.3 ± 6.1	58.9 ± 7.1	66.0 ± 7.8	<0.001
SF	39.6 ± 7.9	39.8 ± 7.2	37.3 ± 8.3	0.081	50.7 ± 9.3	57.8 ± 10.9	63.0 ± 7.6	<0.001
RE	38.0 ± 7.6	37.2 ± 7.8	37.8 ± 7.6	0.798	56.7 ± 10.1	62.8 ± 9.3	63.8 ± 7.3	<0.001
MH	37.6 ± 9.6	36.6 ± 8.6	38.7 ± 8.6	0.332	73.1 ± 8.6	75.2 ± 5.8	83.7 ± 4.7	<0.001
Total score	38.1 ± 3.6	37.3 ± 3.0	37.5 ± 3.2	0.297	54.3 ± 3.6	61.3 ± 2.9	65.6 ± 1.7	<0.001

PF, physical function; RP, role physical; BP, bodily pain; GH, general health; VT, vitality; SF, social function; RE, role of emotion; MH, mental health.

### 4.5 Complications

There were no serious adverse events over 6 months in either group. We found that 3 (4.1%) patients in the RFA + MT group had adverse events within 1 month. Two patients experienced exacerbated pain following manual therapy. One patient demonstrated a reduction in back muscle strength. These complications disappeared at 1 month's follow-up. Four patients experienced exacerbated pain in MT group. The complications disappeared at 1 month's follow-up. While in the RFA group, one patient developed localized hypoesthesia, which subsided on its own by the 1 month's follow-up.

## 5 Discussion

Lumbar facet joint pain is a disease that can severely and comprehensively impair various aspects of quality of life. Patients with lumbar facet joint pain experience moderate to severe pain intensity. Due to the prevalence of lumbar facet joint pain, pain control and quality of life improvement are considered the most important outcome measures when evaluating treatment options (6).

The management of lumbar facet joint pain has remained a clinical challenge. Various therapeutic approaches including nonsteroidal anti-inflammatory drugs (NSAIDs), lifestyle modifications, nerve blocks, and corticosteroid injections have been employed in clinical practice for treating lumbar facet joint pain, yet these interventions continue to be subjects of ongoing debate within the medical community (17).

While the RFA + MT group demonstrated statistically significant improvements across all outcomes, the clinical meaningfulness of these differences should be interpreted in the context of established minimal clinically important differences (MCID). For VAS in chronic low back pain, an MCID of 1.5–2.0 points has been widely reported; the RFA + MT group achieved a mean reduction of 4.1 points from baseline to 6 months, substantially exceeding this threshold. For ODI, the MCID is approximately 10–12 points; the RFA + MT group demonstrated a 12.4-point reduction (from 31.4 to 19.0), meeting or exceeding the MCID. For RMQ, an MCID of 2–3 points has been suggested; the RFA + MT group achieved a 6.9-point improvement (from 15.1 to 8.2), well above the MCID. Furthermore, the SF-36 total score improvement of 28.1 points (from 37.5 to 65.6) in the RFA + MT group far exceeds the MCID of 5–10 points commonly reported for this instrument in chronic pain populations. These findings indicate that the observed differences are not only statistically significant but also clinically meaningful, representing tangible improvements in pain, function, and quality of life that are perceptible and important to patients.

Our results show that compared to RFA alone, RFA combined with manual therapy (RFA + MT) was associated with lower VAS scores at the one-month follow-up. This suggests that combined treatment may be associated with better outcomes in pain management. This association continues in the three and six-month follow-ups. The VAS scores for the RFA + MT group were 3.3 ± 0.6 and 3.2 ± 0.6, while the MT group had 5.7 ± 0.8 and 5.6 ± 0.8, the RFA group had 3.7 ± 0.8 and 3.6 ± 0.8. This suggests the combined therapy may be associated with greater pain relief in both the short and long term. This might be because manual therapy improves joint mobility and reduces muscle tension, enhancing the analgesic effect of RFA for more comprehensive pain control. This study demonstrates that both RFA and MT effectively alleviate pain. Compared to MT or RFA monotherapy, the RFA + MT combination group demonstrated significantly lower VAS scores at the 1-month postoperative follow-up. These findings suggest that the combined treatment protocol exhibits marked clinical superiority in pain management.

In terms of functional recovery, the RFA + MT group also showed superiority in assessments using the Roland-Morris Questionnaire (RMQ) and Oswestry Disability Index (ODI). Especially at three and six months post-surgery, the RFA + MT group had significantly lower RMQ and ODI scores than the MT group and RFA group. This shows the combined therapy may be associated with eases pain and improves lumbar function, potentially enhancing patients' ability to perform daily activities. This potential synergistic association on function recovery may be explained by RFA relieving pain, potentially enabling better patient participation in manual therapy. Then, manual therapy optimizes lumbar biomechanical function, reducing facet joint stress and promoting recovery.

Ford et al.'s (9) study emphasizes the significance of individualized treatment. Their IMT program, designed according to patients' specific pathoanatomy, psychosocial and neurophysiological impairments, optimizes interventions through clinical reasoning processes such as Maitland assessment techniques. This is akin to the “targeted joint mobilization and soft tissue release” strategy in this study's MT approach. Both studies indicate that individualized treatment can break the chronic pain—disability cycle by reducing facet joint stress and enhancing local motor control, as observed in the lumbar deep muscle activation in Ford's study. Moreover, Ford et al. found significant improvements in mental health (Örebro score) and quality of life (EuroQol—5D) in the IMT group. This is consistent with the combined therapy group's notable performance in the SF—36 MH dimension (83.7 vs.75.2, *p* < 0.001) in this study, suggesting that manual therapy may alleviate pain—related psychological burdens through mind—body integration.

From a neurophysiological perspective, RFA selectively destroys the nociceptive signal transmission of the medial branch nerves innervating the lumbar facet joints through thermal energy, providing direct analgesia (18, 19). Manual therapy, on the other hand, stimulates mechanoreceptors and nociceptors in muscles and joints, activates inhibitory neural pathways in the spinal cord and brainstem, and regulates pain signal transmission (20–22). This potential synergistic neurophysiological mechanism may explain why the RFA + MT combination therapy was associated with better outcomes in pain relief than single therapy. Moreover, manual therapy may enhance RFA's long-term efficacy by improving local blood circulation and promoting the clearance of metabolic products, thereby achieving more sustained pain control (23–25).

It should be noted that although RFA is effective for 60%–80% of patients within 6–12 months, its efficacy diminishes over time. This study suggests that the RFA + MT combination therapy may be associated with prolonged RFA's efficacy by addressing underlying biomechanical issues, potentially offering more sustained pain relief. Additionally, patient satisfaction with the combination therapy was higher, likely due to significant pain reduction and functional improvement. Higher satisfaction improves treatment adherence and may positively impact long-term outcomes.

Despite providing evidence for the efficacy of RFA + MT in treating lumbar facet joint pain, this study has several limitations. First, this study is a retrospective observational investigation, which may be susceptible to potential biases such as selection bias and confounding factors. Treatment assignment was determined by clinical decision-making rather than randomization, and may have been influenced by disease severity, patient preferences, and clinician experience. Although we minimized these risks through strict inclusion, exclusion criteria and statistical adjustments, the inherent limitations of retrospective and non-randomized designs may still influence the interpretation of results. Second, we acknowledge that the absence of multivariable-adjusted analyses and propensity score methods is a significant limitation. Even though baseline characteristics appeared comparable, unadjusted comparisons cannot exclude residual confounding from factors such as psychological status, socioeconomic background, disease severity, or clinician-related variables. Future prospective studies should incorporate these analytical approaches or, preferably, randomized designs to strengthen causal inference. Third, the analytical approach was constrained by the retrospective study design, which precluded the use of advanced longitudinal modeling techniques that would optimally account for the correlation structure of repeated measurements. Future prospective studies should incorporate repeated-measures frameworks such as linear mixed-effects models to appropriately model longitudinal data and strengthen causal inference. Fourth, this study excluded patients older than 60 years and those with BMI > 34 kg/m², which limits generalizability to broader populations affected by this condition. Future research should establish clear guidelines for MT techniques to ensure consistency across studies. The sample included a wide age range of patients, but no stratified analysis was conducted to assess the impact of age on treatment efficacy. Older patients may have degenerative comorbidities that could confound the results. Future studies should consider age stratification to better understand how different age groups respond to treatment. Fifth, the 6-month follow-up period represents a significant limitation, particularly for radiofrequency ablation interventions. Published evidence indicates that RFA typically provides sustained analgesia for 6–12 months before potential nerve regeneration leads to diminished efficacy; therefore, our 6-month endpoint may not capture the full duration of RFA effect or the potential for late recurrence (13, 26, 27). The optimal assessment of combined RFA + MT therapy requires follow-up extending to 12 and 24 months to determine whether the observed benefits persist, whether repeat interventions become necessary, and whether manual therapy truly prolongs RFA efficacy as hypothesized. This limitation should be considered when interpreting the sustained benefit of the combination therapy. Finally, although manual therapy was standardized by a single experienced surgeon, future research should establish clear, reproducible guidelines for MT techniques across multiple centers to enhance external validity. Future research can also explore the effects of different manual techniques combined with RFA and optimize individualized treatment plans to enhance therapeutic outcomes. Future research directions could include conducting multi-center RCTs with follow—up extended to 12–24 months to clarify the long- term benefits of combined therapy. Dose—response relationships of MT should be explored, such as the impact of different manual techniques (dynamic joint mobilization vs. soft tissue release) on effectiveness. Additionally, biomechanical improvements of facet joints can be assessed using imaging (e.g., dynamic MRI scans) to provide objective evidence for mechanistic studies.

## 6 Conclusion

In conclusion, the combination of radiofrequency ablation and manual therapy was associated with significantly greater improvements compared to either treatment alone in reducing pain, improving functional capacity, and enhancing the quality of life in patients with chronic lumbar facet joint pain over a 6-month period.

## Data Availability

The original contributions presented in the study are included in the article/Supplementary Material, further inquiries can be directed to the corresponding author.
